# Long Noncoding RNAs in Diet-Induced Metabolic Diseases

**DOI:** 10.3390/ijms25115678

**Published:** 2024-05-23

**Authors:** Annette Brandt, Florian Kopp

**Affiliations:** 1 Molecular Nutritional Science, Department of Nutritional Sciences, University of Vienna, 1090 Vienna, Austria; annette.brandt@univie.ac.at; 2 Clinical Pharmacy Group, Department of Pharmaceutical Sciences, University of Vienna, 1090 Vienna, Austria

**Keywords:** lncRNA, long noncoding RNA, metabolic disease, hepatic steatosis, MASLD, NAFLD, type 2 diabetes, obesity

## Abstract

The prevalence of metabolic diseases, including type 2 diabetes and metabolic dysfunction-associated steatotic liver disease (MASLD), is steadily increasing. Although many risk factors, such as obesity, insulin resistance, or hyperlipidemia, as well as several metabolic gene programs that contribute to the development of metabolic diseases are known, the underlying molecular mechanisms of these processes are still not fully understood. In recent years, it has become evident that not only protein-coding genes, but also noncoding genes, including a class of noncoding transcripts referred to as long noncoding RNAs (lncRNAs), play key roles in diet-induced metabolic disorders. Here, we provide an overview of selected lncRNA genes whose direct involvement in the development of diet-induced metabolic dysfunctions has been experimentally demonstrated in suitable in vivo mouse models. We further summarize and discuss the associated molecular modes of action for each lncRNA in the respective metabolic disease context. This overview provides examples of lncRNAs with well-established functions in diet-induced metabolic diseases, highlighting the need for appropriate in vivo models and rigorous molecular analyses to assign clear biological functions to lncRNAs.

## 1. Introduction

Based on current data from the Global Burden of Disease Study 2019, the worldwide prevalence of metabolic diseases, referring to metabolic dysregulated processes such as type 2 diabetes (T2D) or metabolic dysfunction-associated steatotic liver disease (MASLD), is still growing [1]. While the estimated worldwide prevalence of MASLD is ~32.4% [2], ~529 million people worldwide suffered from T2D in 2021 [3], contributing to a high economic burden [4,5]. The main risk factors and metabolic comorbidities of MASLD or T2D include obesity, insulin resistance, hyperlipidemia, or high blood pressure [6,7]. Indeed, data from population-based studies suggest that ~878 million people suffered from obesity in 2022 [8]. Besides genetic factors and a low physical activity, general overnutrition as well as the intake of certain macronutrients is associated with obesity, MASLD, or T2D [9,10]. Indeed, a Westernized diet, rich in monosaccharides like fructose and fats that are rich in short-chain fatty acids (SFAs), is associated with the development of MASLD [9] and T2D [11]. However, the molecular mechanisms contributing to diet-induced metabolic dysregulated processes are, so far, not fully understood.

In recent years, noncoding RNAs have been increasingly implicated in the response to different nutritional conditions and in diet-induced metabolic diseases [12,13]. While the molecular functions of microRNAs, small ~22 nucleotide (nt)-long regulatory RNAs that are known to post-transcriptionally repress target mRNAs to which they bind [14], are well established (also in the context of metabolic diseases [15]), relatively little is known about the precise molecular roles of another class of noncoding RNAs termed long noncoding RNAs (lncRNAs). lncRNAs are generally defined by a sequence length of more than 200 nt and by the lack of a protein-coding open reading frame [16]. To better distinguish lncRNA genes from medium-sized ones—mostly RNA polymerase III-transcribed RNAs—a recently published consensus statement recommends a different, slightly bigger cut-off size of 500 nt for lncRNA genes [17]. However, irrespective of the precise definition of their length, lncRNAs comprise a very heterogenous group of noncoding transcripts not only in terms of their genomic location and biogenesis but also in terms of their biological and molecular functions. lncRNAs are mostly transcribed by polymerase II, often spliced and polyadenylated. Some of them exist as circularized transcripts that are produced by the back-splicing of certain coding as well as noncoding RNAs [18]. They can originate from intergenic regions or overlap with other coding or noncoding genes in either the sense or the antisense orientation. In addition, active DNA elements are often transcribed, resulting in the production of enhancer- or promoter-derived lncRNA transcripts [16,17].

While ~75% of the human genome is transcribed into RNAs, with most of them representing noncoding RNAs with tens of thousands being annotated as lncRNAs [19,20], the physiological and molecular roles of the vast majority of these noncoding transcripts are still unknown [21]. Nevertheless, lncRNA functions can be generally categorized into two modes: (1) the transcriptional regulation of neighboring gene loci in the nucleus, and (2) the modulation of numerous biological processes at various cellular locations and compartments that are distant to the lncRNA transcription site. On a molecular level, lncRNAs have been shown to interact with other RNA, DNA, or protein molecules to control their stability, activity, or subcellular localization in distinct cellular compartments. Some of them have been also demonstrated to encode microproteins or peptides that can exert important cellular functions [22,23]. Of note, each of these modes of action needs to be experimentally determined on a case-by-case basis for each lncRNA candidate gene to imply meaningful biological functions for the encoded transcript [21]. So far, several critical roles have been described for lncRNAs in normal physiology, including, for example, development and stem cell function [24,25], as well as in pathophysiological disease conditions, such as neurological disorders [26], cancer [27] or cardiac disease [28].

While lncRNAs have also been shown to be dysregulated in models of metabolic liver disease, such as MASLD [29] (and a few lncRNAs have been suggested as playing a role in nutritional response and metabolic pathways [13]), clear proof of their functional relevance and molecular modes of action in their respective diet and disease contexts remains missing for many, if not most, of the lncRNA genes. Here, we aim to provide a detailed overview of selected lncRNAs with established functions in diet-induced metabolic disorders that have been proven and validated in suitable in vivo models, i.e., in genetic mouse models with lncRNA loss and gain of function. This review, therefore, does not represent a summary of all lncRNAs that have been published in the context of diet-related metabolic diseases but rather a selection of lncRNA studies that have used appropriate lncRNA knockout and transgenic mouse models to demonstrate a clear functional relationship between an lncRNA and a diet-induced phenotype in mice.

## 2. Adipose Tissue-Related lncRNAs and Diet-Induced Obesity

Weight gain in obesity mainly results from energy intake that exceeds energy expenditure [30]. This positive energy balance is supported by excess dietary energy intake and reduced physical activity [30]. A Western dietary pattern especially contributes to adipose tissue dysfunction and is associated with obesity and related metabolic dysfunction [31]. While white adipose tissue (WAT) is able to store excess energy, brown fat thermogenesis in brown adipose tissue (BAT) contributes to energy expenditure in form of thermogenesis [32], and studies suggest that the browning of WAT might protect against obesity and associated metabolic diseases [33].

**Figure 1:**
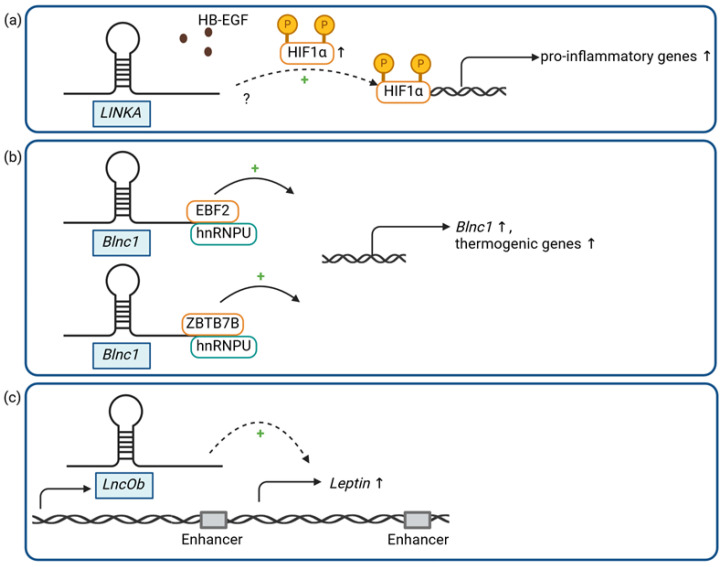
Adipose-tissue-related lncRNAs and diet-induced obesity: (**a**) *LINKA*, (**b**) *Blnc1*, and (**c**) *LncOb* (P = phosphorylation, ↑ = increase, ↓ = decrease, ? = unclear mechanism). Figure created with BioRender.com.

Several studies suggest that lncRNAs are associated with white adipocyte differentiation [34,35,36,37]. Long intergenic non-coding RNA for kinase activation (*LINKA*, also known as *LINK-A* or *LINC01139*), an approximately 1.5 kilobase (kb)-long and predominantly cytoplasmic intergenic lncRNA, has been initially described as a prognostic lncRNA that positively regulates hypoxia-inducible factor 1 alpha (HIF1α) signaling in triple negative breast cancer [38]. Mechanistically, *LINKA* facilitates the heparin-binding (HB)-EGF-mediated recruitment of two kinases, breast tumor kinase (BRK) and leucin-rich repeat kinase 2 (LRRK2), to the EGFR-GPNMB (epidermal growth factor receptor–glycoprotein NMB) dimer, which results in the phosphorylation of HIF1α at two sites, leading to HIF1α stabilization and activation under normoxic conditions [38]. Recently, *LINKA* has also been shown to be a human-specific lncRNA that is induced in the breast tissue, subcutaneous WAT, and visceral adipose tissue of overweight patients and whose expression correlates with increasing body mass indices [39]. In a mouse model, in which human *LINKA* had been knocked-in into the mouse genome, *LINKA* overexpression promoted high-fat-diet (HFD)-induced obesity and insulin resistance [39]. Moreover, mice with *LINKA* overexpression suffer from inflammatory alterations in adipose tissue as well as mammary glands. *LINKA* promotes the production of pro-inflammatory factors in mouse mammary glands via stabilization of the transcription factor HIF1α, which, in turn, transcriptionally activates inflammatory genes, such as interleukin 1 beta (*Il1b*) and C-X-C motif chemokine ligand 16 (*Cxcl16*). This is associated with suppressed energy expenditure and a reduced adaptive thermogenic function in mice, as demonstrated by a significant reduction in uncoupling protein 1 (*Ucp1*), which facilitates heat generation in the mitochondria of BAT and related thermogenesis genes in several adipose tissues after cold exposure [39]. On a molecular level, *LINKA* cooperates with HB-EGF to stabilize HIF1α (Figure 1a), potentially via a similar mechanism previously described in human breast cancer cells [38].

*Blnc1* (brown fat lncRNA 1, also known as *AK038898*) was originally discovered as an approximately 1 kb long conserved murine intergenic lncRNA that is critical for brown adipogenesis [40]. *Blnc1* is induced during brown and beige adipocyte differentiation and is required for the induction of thermogenic gene expression, including the *Ucp1* gene (Figure 1b). *Blnc1*, a primarily nuclear lncRNA, has been proposed to form a ribonucleoprotein complex with the EBF transcription factor 2 (EBF2), an important regulator of BAT, to drive the thermogenic phenotype [40]. Interestingly, *Blnc1* itself is a target of EBF2, and both *Blnc1* and EBF2 are required for efficient brown adipogenesis, suggesting that *Blnc1*–EBF2 cooperatively regulate thermogenesis in brown fat. In a later study [41], *Blnc1* was found to be conserved in both humans and mice, also sharing its function in brown adipogenesis. Moreover, a conserved RNA domain was identified in human and mouse *Blnc1* that is critical for brown adipocyte differentiation and thermogenesis. This RNA domain has been reported to bind to heterogeneous nuclear ribonucleoprotein U (HNRNPU), a ubiquitously expressed RNA binding protein, which enables the formation of the *Blnc1*–EBF2 complex and, hence, the activation of the thermogenic program [41]. In addition, HNRNPU has been reported to recruit *Blnc1* into another ribonucleoprotein complex containing the transcription factor ZBTB7B (zinc finger and BTB domain-containing protein 7), a potent driver of brown adipogenesis and thermogenesis [42]. ZBTB7B has been shown to be required for the adaptation to the cold and the induction of a thermogenic gene program. Interestingly, in both the genetic and HFD-induced mouse models of obesity, *Blnc1* expression was significantly increased in epididymal WAT (eWAT) and BAT, positively correlated with body weight, and induced in cold-induced thermogenesis [43]. Moreover, Zhao et al. demonstrated that transgenic mice with adipocyte-specific *Blnc1* inactivation exacerbated HFD-induced insulin resistance and hepatic steatosis, while body weight gain was not significantly different between control and knockout mice [43]. However, knockout mice showed increased brown fat whitening and adipose tissue inflammation during HFD feeding, while *Ucp1* mRNA expression and further markers of thermogenesis were reduced in these mice compared to HFD-fed controls [43]. On the contrary, a fat-specific transgenic expression of *Blnc1* protected mice from HFD-induced obesity, adipose tissue dysfunction, and metabolic dysfunction like insulin resistance and hepatic steatosis [43,44].

The peptide hormone leptin is able to regulate food intake and body mass and seems to be upregulated in obese patients, which suggests that these patients suffer from leptin resistance [45]. Moreover, serum leptin concentrations correlate with the amount of body fat [46]. While leptin is also produced by other tissues such as the stomach [47], two leptin enhancer sequences up- and downstream of the leptin gene have been identified as necessary for fat-specific leptin expression in mice. Moreover, it has been verified that the proximal leptin promoter sequence is regulated by the two leptin enhancers and is required for robust leptin expression [48]. In addition to the identification of two transcription factors (EBF transcription factor 1/EBF1 and nuclear factor 1/NF1) that bind to the leptin enhancers and promoter, several RNA binding proteins have been also detected to be enriched at the leptin promoter, suggesting that RNAs may be involved in the regulation of leptin expression as well. Indeed, one 9 kb long lncRNA is co-transcribed with leptin from a region further upstream of the upstream leptin enhancer. This lncRNA, also called long noncoding RNA upstream of leptin (*Lnclep* or *LncOb*), is spliced to an ~850 nt long mature lncRNA that shows considerable conservation in humans. *LncOb* is only expressed in adipose tissue, is co-regulated with leptin, and does not bind to leptin enhancers (Figure 1c). It rather binds to the leptin proximal promoter and interacts with RNA binding proteins identified to be enriched at the leptin promoter [48]. *LncOb* is highly correlated with leptin mRNA in HFD-induced obese mice and exclusively expressed in inguinal WAT and eWAT [48]. Like in mice, RNA corresponding to *LncOb* in humans is expressed in adipose tissue and is correlated with leptin mRNA, while it is not expressed in liver or muscle tissue. Remarkably, knockout of *LncOb* in mice was associated with an accelerated body weight gain and dysregulated leptin expression under HFD as compared to wild-type control mice [48]. This phenotype could be reverted by an infusion of the leptin hormone into diet-induced obese mice. Of note, a single-nucleotide polymorphism has been identified in the human *LncOb* gene that correlates with decreased leptin expression and obesity in humans [48]. Although these findings clearly point to a leptin-dependent function of *LncOb* in fat metabolism and adipose tissue homeostasis, the precise mechanism of how this lncRNA confers transcriptional regulation of the leptin locus remains an interesting open question.

## 3. LncRNAs and Diet-Induced Hepatic Steatosis

Adipose tissue dysfunction results in an impaired ability to store fat and has therefore been linked to the pathogenesis of hepatic steatosis [49]. The hallmark of hepatic steatosis is the excessive accumulation of triglycerides in hepatocytes resulting from esterification of free fatty acids (FFAs) in the liver. These FFAs can originate from various sources, such as (1) release of FFAs from increased lipolysis in adipose tissue that reach the liver via the blood, (2) FFAs arising from de novo lipogenesis (DNL) from carbohydrates, and (3) from the intake of dietary fats that reach the liver via FFAs in the blood or via chylomicron remnants [50,51]. In principle, triglycerides can be excreted from the liver as very-low-density lipoprotein (VLDL), stored in lipid droplets in the liver or metabolized as FFAs via β-oxidation [51,52,53].

### 3.1. LncRNAs and Lipogenesis

**Figure 2:**
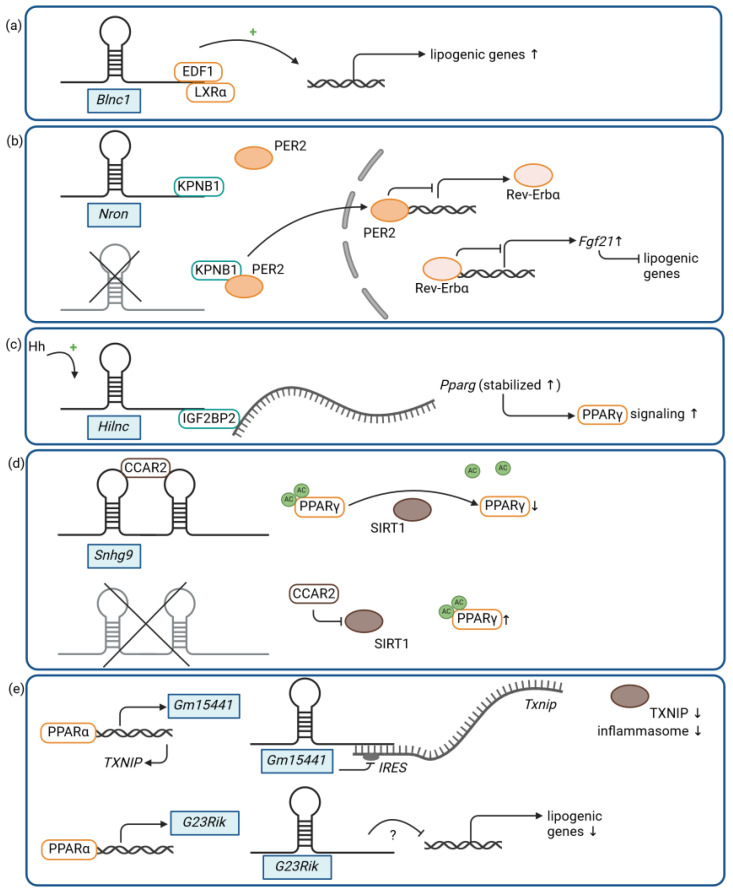
LncRNAs and diet-induced hepatic steatosis: (**a**) *Blnc1*, (**b**) *Nron*, (**c**) *Hilnc*, (**d**) *Snhg9*, and (**e**) *Gm15441* and *G23Rik* (Ac = acetylation, ↑ = increase, ↓ = decrease, ? = unclear mechanism.). Figure created with BioRender.com.

Recent data suggest that several lncRNAs are dysregulated in MASLD patients (for an overview, see [54,55]), and studies report that lncRNAs are involved in lipogenesis and fatty acid metabolism (for an overview, see [56]). Indeed, *Blnc1* function is not restricted to the adipose tissue, as it has been shown to regulate lipogenesis in the liver as well [57]. As demonstrated by Zhao et al., *Blnc1* and *Srebp1c* (sterol regulatory element-binding transcription factor 1c), a gene known to regulate the expression of genes involved in DNL and triglyceride synthesis, are similarly induced in the livers of HFD-fed mice, and *Blnc1* expression correlates with body weight and liver triglyceride content [57]. A liver-specific *Blnc1* knockout model attenuates HFD-induced weight gain, hepatic steatosis, and insulin resistance in mice, resulting in protection from MASLD. As in its adipose-specific functions, *Blnc1* appears to facilitate the formation of a distinct ribonucleoprotein transcriptional complex in the liver, consisting of *Blnc1*, endothelial differentiation-related factor 1 (EDF1), and liver X receptor alpha (LXRα), which drives lipogenic gene expression, including the expression of *Srebp1c* (Figure 2a) [57]. These findings demonstrate that a conserved lncRNA can modulate metabolic functions in various tissues and cell types by a similar yet distinct molecular mechanism, which is the formation of different ribonucleoprotein transcriptional complexes for the control of metabolism-associated gene programs.

Another lncRNA, which is not only relevant in adipose tissue but also in the liver, is the lncRNA noncoding repressor of NFAT (*Nron*) [58], a roughly 3 kb long antisense transcript derived from an intron of the protein-coding gene *Mvb12b* (multivesicular body subunit 12B). *Nron* is expressed in various tissues, such as liver, adipose tissue, or muscle tissue. It was initially described as a negative regulator of nuclear transport and, hence, the activity of NFAT (nuclear factor of activated T cells), a class of transcription factors that are responsive to calcium signaling and modulate the immune response in T lymphocytes [59]. *Nron* interacts with nuclear transport factors like KPNB1 (karyopherin subunit beta 1), scaffold proteins like IQGAP1 (IQ motif containing GTPase-activating protein 1), and known NFAT kinases like CK1 (casein kinase 1) to form an RNA-protein scaffold complex that regulates NFAT phosphorylation and subcellular localization [59,60]. Disruption of this complex by either silencing *Nron* or IQGAP1 results in dephosphorylation, nuclear import, and the activation of NFAT [60]. Recently, it has been demonstrated that *Nron* levels decrease in the mouse liver after HFD feeding [58]. In line with these findings, patients with MASLD suffer from decreased *NRON* expression in the liver [58]. Unexpectedly, Liu et al. demonstrated that *Nron* knockout mice were protected from HFD-induced weight gain, obesity, and insulin resistance, and that *Nron* depletion promoted adipocyte lipolysis and lipid turnover [58]. This was associated with an amelioration of HFD-induced hepatic steatosis, while HFD-induced hepatic inflammation and apoptosis were not affected. By contrast, *Nron* overexpression leads to an intensification of the HFD-induced hepatic metabolic changes [58]. Interestingly, the effects observed in *Nron*-deficient animals could not be attributed to an activation of NFAT signaling. In the liver and in isolated hepatocytes, *Nron* loss-of-function mutation promotes the binding of the circadian clock regulator PER2 (period circadian clock 2) to KPNB1, a nucleocytoplasmic transport protein that also interacts with *Nron* [59], resulting in the nuclear translocation of PER2. Nuclear PER2 represses the expression of another circadian clock gene, Rev-Erbα, also known as nuclear receptor subfamily 1 group D member 1 (*Nr1d1*), which in turn activates the expression of fibroblast growth factor 21 (*Fgf21*), a key regulator of energy metabolism in the liver (Figure 2b). Increased FGF21 levels inhibit lipogenesis and induce fatty acid oxidation via an activation of AMPK (AMP-activated protein kinase) signaling, eventually improving hepatic steatosis under HFD. On the contrary, diet-induced effects on the adipose tissue in *Nron* knockout mice, such as attenuated obesity, appear to be independent of the *Nron*–KPNB1–PER2– Rev-Erbα–FGF21 axis [58].

In a screen for lncRNAs regulated by Hedgehog (Hh) signaling, *Gm16364* has been identified as an approximately 800 kb long murine Hh-induced lncRNA, also termed *Hilnc* (Hedgehog signaling-induced long noncoding RNA) [61]. Hh signaling is a deeply conserved signaling pathway regulating development and tissue homeostasis, in which Hh ligands activate their respective receptors and eventually activate the Gli family of transcription factors [62,63]. Hh signaling has also been implicated in metabolic processes in the adipose tissue, as well as in the liver and in metabolic liver diseases [64,65]. While *Hilnc* is directly induced by Hh signaling via Gli transcription factors in various tissues, it was significantly upregulated in liver tissue in a diet-induced mouse model of hepatic steatosis [61]. Remarkably, the depletion of *Hilnc*, either through promoter mutation or the deletion of exons 2 and 3, reduced DNL and protected mice from HFD-induced obesity, impaired glucose metabolism, and hepatic steatosis. On the other hand, adenovirus-mediated overexpression of *Hilnc* in the livers of *Hilnc* knockout mice promoted lipid accumulation under an HFD. On a molecular level, *Hilnc* has been shown to bind via a distinct RNA region to the RNA binding protein IGF2BP2 (insulin-like growth factor 2 mRNA binding protein 2) in the liver and to positively modulate PPARγ (peroxisome proliferator-activated receptor gamma) signaling [61], a central regulator of lipid metabolism [66]. IGF2BP2 regulates the stability and translation of mRNAs to which it binds [67,68], and *Hilnc* is required for the proper function of IGF2BP2 as a post-transcriptional regulator of interacting mRNAs, such as the *Pparg* mRNA (Figure 2c). In the livers of HFD-fed wild-type mice, *Pparg* levels, as well as PPARγ signaling, were induced in a *Hilnc*- and IGF2BP2-dependent manner, affecting lipid metabolism and promoting hepatic steatosis [61]. Of note, while there appears to be no syntenic lncRNA or sequence-based homolog of *Hilnc* in the human genome, a potential functional human homolog, referred to as *h-Hilnc*, has been identified, which is also responsive to Hh signaling, regulates *PPARG* via IGF2BP2, and is involved in lipid metabolism and lipid accumulation in human liver cells [61].

### 3.2. LncRNA and Intestinal Lipid Absorption

Alterations of intestinal microbiota composition and an impaired intestinal barrier function in humans are associated with several metabolic diseases, including diabetes and MASLD [69,70]. Indeed, studies in germ-free mice further indicate that microbiota promotes monosaccharide absorption from intestinal lumen, which contributes to hepatic DNL [71]. Moreover, intestinal microbiota also seems to affect intestinal lipid absorption [72], and lncRNAs might be able to affect microbiome-host-interaction [73]. In line with these findings, small nucleolar RNA host gene 9 (*Snhg9*) has been shown to be significantly lower expressed in small intestines from conventional mice as compared to germ-free or antibiotic-treated mice, suggesting that *Snhg9* expression is decreased in the presence of microbiota [74]. *Snhg9*, an approximately 200 nt lncRNA encoding the small nucleolar RNA 78 (*Snora78*), has been further shown to bind via distinct RNA structures to the cell cycle and apoptosis protein 2 (CCAR2), an endogenous inhibitor of sirtuin 1 (SIRT1), a known repressor of PPARγ activity as well as expression. Accordingly, *Snhg9* sequesters CCAR2, resulting in the release of SIRT1 from CCAR2-mediated inhibition and, consequently, in the SIRT1-mediated repression of PPARγ (Figure 2d) [74]. In line with these findings, *Snhg9* knockout in 3T3-L1 cells increases PPARγ expression and activates PPARγ signaling, thereby suppressing the lipid metabolism, which can be reversed by *Snhg9* overexpression [74]. Likewise, a small intestinal epithelial cell-specific overexpression of *Snhg9* impairs PPARγ expression and the genes involved in fatty acid absorption, transport, and synthesis in mice, resembling the effects seen in germ-free or antibiotic-treated animals. *Snhg9* transgenic mice showed reduced intestinal lipid absorption after being fed an HFD and were therefore also protected from HFD-induced obesity, insulin resistance, and hepatic steatosis when compared to wild-type controls [74]. The physiological suppression of *Snhg9* by microbiota is accomplished via a Toll-like receptor-mediated activation of myeloid cells and subsequent cytokine-triggered signaling to the intestinal epithelial cells, resulting in the intestinal repression of *Snhg9* [74]. Although the benefits of microbiota-mediated regulation of the lipid metabolism for the host organism, as well as the precise components of the microbiota that are necessary for this regulation, are still unknown, these results demonstrate how microbiota can positively influence lipid absorption and fat metabolism by downregulating a noncoding RNA in host intestinal epithelial cells. An intriguing open question is also whether these functions are evolutionarily conserved and whether human *SNHG9* displays the same functions in the regulation of lipid absorption and fat metabolism.

### 3.3. LncRNAs and Metabolic Stress

The PPARα-induced murine lncRNA *Gm15441* is an approximately 1.7 kb long transcript antisense to a protein-coding gene—thioredoxin-interacting protein (*Txnip*)—that consists of four exons, two of which overlap the *Txnip* gene [75]. Upon PPARα activation by either a pharmacological compound or fasting, *Gm15441* is dramatically and specifically increased in the mouse liver. *Gm15441* and its antisense transcript, *Txnip*, are both direct PPARα targets, whereas their regulation upon PPARα activation is inverse. Upon metabolic stress, such as fasting, *Gm15441* is induced in a PPARα-dependent manner in the liver and inhibits *Txnip* translation likely via the interaction of its exon 3 with the overlapping antisense sequence of the 5′ untranslated region of *Txnip*, which harbors internal ribosome entry site (IRES) sequences critical for efficient *Txnip* translation (Figure 2e) [75]. The reduced TXNIP levels result in decreased activation of the NLR family pyrin domain-containing-3-(NLRP3)-mediated inflammasome accompanied by a reduced cleavage of caspase 1 (CASP1) and impaired maturation of IL1B. In a *Gm15441* mouse knockout model, in which the expression of *Gm15441* is specifically impaired while *Txnip* expression is unaffected, *Gm15441* deficiency promoted the activation of the NLRP3-mediated inflammasome as well as lipid accumulation in the liver upon PPARα activation, either through pharmacological intervention or acute fasting [75]. Interestingly, there is another murine lncRNA, called *3930402G23Rik* (or *G23Rik*), that has been shown to also be induced by PPARα signaling specifically in the liver and to modulate hepatic liver metabolism [76]. Acute fasting in *G23Rik*-deficent mice potentiated hepatic lipid accumulation accompanied by an increase in cholesterol and triglyceride levels [76]. Although the molecular mechanism of how *G23Rik* contributes to the regulation of the PPARα-mediated lipid metabolism is unknown, the two examples, *Gm15441* and *G23Rik*, demonstrate how lncRNAs can respond to metabolic stress and modulate critical metabolic pathways, such as PPAR signaling.

## 4. LncRNA and Glucose Metabolism

Insulin resistance is often a common characteristic of obesity and diet-induced metabolic diseases, such as T2D or MASLD [77]. While a dysregulated lncRNA profile seems to be involved in the pathogenesis of diet-induced metabolic diseases such as MASLD [54,55], recent evidence suggests that lncRNAs also contribute to the regulation of insulin signaling and glucose metabolism in several tissues and to the pathogenesis of insulin resistance (reviewed in detail by others [78,79]). By controlling pathways of glucose metabolism, the liver has a major role in the control of glucose homeostasis [80], and insulin resistance seems to be associated with an increase in hepatic glucose output, lipogenesis, and VLDL secretion, leading to a combination of hyperglycemia and hypertriglyceridemia [81].

**Figure 3:**
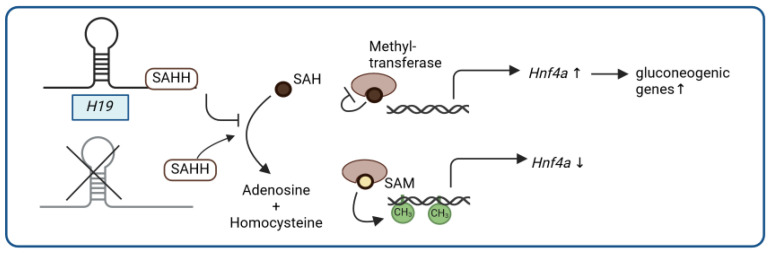
LncRNA and glucose metabolism: *H19* (CH3 = methylation, ↑ = increase, ↓ = decrease). Figure created with BioRender.com.

Noncoding RNAs, such as the lncRNA *H19*, have been described as performing key roles in liver biology and disease [82]. *H19* is known as a ~2.3 kb long paternally imprinted lncRNA originating from the insulin growth factor 2 (*IGF2*/*Igf2*) locus in humans and mice [83]. On the maternal allele, *H19* is expressed while *Igf2* expression is prevented; this reciprocal regulation of *H19* and *Igf2* is important during fetal development. After birth, *H19* is downregulated in most tissues, except in the skeletal muscle and heart [83]. *H19* expression has been furthermore shown to be increased in the livers of T2D patients [84], and, consistent with *H19* expression in T2D patients, *H19* levels are also increased in livers of mice with diet-induced impaired glucose metabolism [85]. In line with these findings, the liver-specific overexpression of *H19* results in impaired glucose homeostasis with hyperglycemia and insulin resistance, whereas a whole-body knockout of *H19* in mice leads to improved insulin sensitivity and reduced insulin-dependent endogenous glucose production in the liver, associated with the reduced expression of gluconeogenic genes [85]. On a molecular level, *H19* negatively regulates the promoter methylation and, hence, activates the expression of the hepatocyte nuclear factor 4α (*Hnf4a*), a transcription factor important for gluconeogenesis. As previously reported for other tissues [86], *H19* binds to S-adenosylhomocysteine hydrolase (SAHH) in mouse liver as well [85]. SAHH hydrolyzes S-adenosylhomocysteine (SAH), an inhibitor of S-adenosylmethionine (SAM)-dependent methyltransferases, which results in the activation of DNA methyltransferases and, consequently, results in increased DNA promoter methylation. It has been suggested that *H19* may cause *Hnf4a* promoter hypomethylation via the *H19*/SAHH pathway through binding to SAHH, as well as the subsequent accumulation of SAH and inhibition of DNA methyltransferases (Figure 3) [85]. In line with these findings, temporally increased *H19* levels upon fasting or chronically elevated *H19* levels reduce *Hnf4a* promoter methylation and induce a gluconeogenic gene expression program. It is noteworthy that previously established modes of action for *H19*, including *H19*-encoded *miR-675* or *Igf2* expression, appear to be dispensable for this metabolic function [85]. Moreover, *H19* has been implicated as contributing to a range of metabolic diseases, such as diet-induced hepatic steatosis, although the underlying molecular mechanisms are less well understood and require more investigation [87,88,89,90,91].

## 5. Conclusions

**Figure 4:**
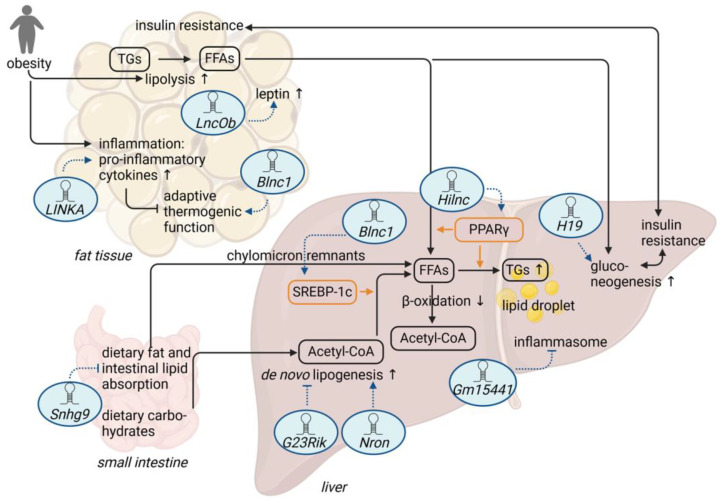
Summary of discussed lncRNAs and their roles in diet-induced obesity, hepatic steatosis, and insulin resistance. (FFAs = free fatty acids, TGs = triglycerides, ↑ = increase, ↓ = decrease) Figure created with BioRender.com.

As summarized in this review, several studies suggest that lncRNAs are important regulatory factors in the pathogenesis of obesity and diet-induced metabolic diseases, such as MASLD or T2D (Figure 4, Table 1). Indeed, data from patients, as well as animal models, indicate tissue-specific regulation of lncRNAs in diet-associated metabolic diseases. Still, the functional roles of the discussed lncRNAs and their relationship with obesity, MASLD, or T2D were mainly assessed in preclinical studies and need to be translated to human pathophysiology. The lncRNAs are involved in different metabolic pathways, including adipocyte differentiation, lipogenesis, or gluconeogenesis. While some lncRNAs are related to metabolic stress during fasting, others respond to dietary changes. Importantly, some lncRNAs like *Blnc1* have functional relevance in several tissues, such as adipose tissue and the liver, acting on several metabolic pathways. Additionally, these lncRNAs might also have the potential to serve as biomarkers for the progression and management of diet-induced metabolic diseases [55,92]. Indeed, *H19* or *NRON* expression is altered in patients with T2D or MASLD [58,84].

On a cellular level, lncRNAs have been shown to act via diverse molecular modes of action to respond to nutritional conditions and to modulate diet-induced metabolic diseases (Figure 4). lncRNAs can thereby regulate transcription of key metabolic genes, either directly as part of a larger transcriptional ribonucleoprotein complex (*Blnc1*) or indirectly via the regulation of the stability (*LINKA*) or activity and expression (*Snhg9*) of metabolism-related transcription factors, such as HIF1α or PPARγ, respectively. lncRNAs like *H19* can also cause promoter hypomethylation of gluconeogenic genes in the liver via binding to and inhibition of the SAH-converting enzyme SAHH in liver cells, leading to an inhibition of SAM-dependent DNA methyltransferases. Another possible control mechanism of key metabolic pathways, such as PPARγ, is the lncRNA-mediated and IGF2BP2-dependent stabilization of *Pparg* mRNA, resulting in increased PPARγ signaling, as demonstrated for *Hilnc*. In addition, lncRNAs can affect the translation of target proteins as antisense transcripts that block, for example, IRES sequences of a target mRNA, such as the lncRNA *Gm15441* and the *Txnip* mRNA, which results in the inhibition of the inflammasome in the liver. Besides protein stability and expression, lncRNAs may also affect the subcellular localization of target proteins critical for metabolic diseases, as exemplified by the *Nron*–KPNB1 interaction, which prevents the nuclear translocation of the circadian clock protein PER2 and subsequently promotes the expression of *Fgf21* and lipogenic genes.

In summary, these selected examples of lncRNAs with distinct roles in diet-induced metabolic diseases demonstrate the necessity of applying appropriate genetic and transgenic mouse models to study the physiological function of new candidate lncRNA genes in diet-dependent disease contexts. Moreover, rigorous testing of the molecular modes of action of selected lncRNA genes further bolsters the functional relevance of the respective lncRNA transcripts in response to nutritional challenges and metabolic diseases. Our selection of lncRNAs described in this review is not supposed to be a comprehensive summary of all diet- and nutrition-associated lncRNAs. Rather, we chose a set of lncRNAs that underwent rigorous testing of their physiological in vivo functions, as well as their molecular modes of action. We foresee that there will be more lncRNAs with important functions in nutrition, metabolism, and metabolic disease that can be uncovered by future studies and that can broaden our understanding of the role of the noncoding transcriptome in metabolic pathways and disease.

## Figures and Tables

**Table 1:** Summary of discussed lncRNAs.

LncRNA	Regulatory Mechanism	Role in Diet-Associated Metabolic Disorders
*LINKA*	HB-EGF-mediated stabilization of the transcription factor HIF1α and induction of pro-inflammatory genes	*LINKA* overexpression + HFD: ↑ obesity; ↑ insulin resistance [39]
*Blnc1*	formation of a ribonucleoprotein transcriptional complex with EBF2 and ZBTB7B via HNRNPU and induction of thermogenic genes	adipocyte-specific *Blnc1* knockout + HFD: ↑ insulin resistance; ↑ hepatic steatosis fat-specific transgenic expression of *Blnc1* + HFD: ↓ adipose tissue dysfunction; ↓ insulin resistance; ↓ hepatic steatosis [43]
*Blnc1*	formation of a ribonucleoprotein transcriptional complex with EDF1 and LXRα and induction of lipogenic genes	liver-specific *Blnc1* knockout + HFD: ↓ weight gain; ↓ hepatic steatosis; ↓ insulin resistance [57]
*LncOb*	binding to the leptin promoter and co-regulation of leptin expression	*LncOb* knockout + HFD: ↑ body weight gain [48]
*Nron*	binding to KPNB1 and negative regulation of the nuclear translocation of the transcriptional repressor PER2, indirectly resulting in the repression of *Fgf21* expression and increased lipogenesis	*Nron* knockout + HFD: ↓ obesity; ↓ insulin resistance; ↓ hepatic steatosis [58]
*Hilnc*	binding to IGF2BP2 and positive modulation of PPARγ signaling	*Hilnc* knockout + HFD: ↓ obesity; ↓ impaired glucose metabolism; ↓ hepatic steatosis [61]
*Snhg9*	binding to CCAR2, resulting in SIRT1-mediated repression of PPARγ signaling	intestinal epithelial cell-specific transgenic expression of *Snhg9* + HFD: ↓ obesity; ↓ insulin resistance; ↓ hepatic steatosis [74]
*Gm15441*	antisense transcript to the protein-coding gene *Txnip*, resulting in the inhibition of *Txnip* translation and the TXNIP-mediated inflammasome	*Gm15441* knockout + acute fasting: ↑ hepatic steatosis [75]
*G23Rik*	inhibition of lipogenic genes via an unknown mechanism	*G23Rik* knockout + acute fasting: ↑ hepatic steatosis [76]
*H19*	binding to SAHH leading to promoter hypomethylation and activation of a gluconeogenetic gene program	*H19* knockout: ↑ insulin-dependent suppression of hepatic glucose production liver-specific overexpression of *H19*: ↑ hyperglycemia; ↑ insulin resistance [85]

↑ = increase, ↓ = decrease.

## Abbreviations

Ac—Acetylation, AMPK—AMP-activated protein kinase, BAT—brown adipose tissue, Blnc1—brown fat lncRNA 1, BRK—breast tumor kinase, CASP1—caspase 1, CCAR2—cell cycle and apoptosis protein 2, CK1—casein kinase 1, Cxcl16—C-X-C motif chemokine ligand 16, DNL—de novo lipogenesis, EBF2—EBF transcription factor 2, EDF1—endothelial differentiation-related factor 1, EGFR-GPNMB—epidermal growth factor receptor–glycoprotein NMB, eWAT—epididymal WAT, FFAs—free fatty acids, Fgf21—fibroblast growth factor 21, G23Rik—3930402G23Rik, HFD—high-fat diet, Hh—Hedgehog, HIF1α—hypoxia-inducible factor 1 alpha, Hilnc—Hedgehog signaling-induced long noncoding RNA, Hnf4a—hepatocyte nuclear factor 4α, HNRNPU—heterogeneous nuclear ribonucleoprotein U, IGF2—insulin growth factor 2, IGF2BP2—insulin-like growth factor 2 mRNA binding protein 2, IL1B—interleukin 1 beta, IRES—internal ribosome entry site, IQGAP1—IQ motif containing GTPase-activating protein 1, kb—kilobases, KPNB1—karyopherin subunit beta 1, LINKA—long intergenic noncoding RNA for kinase activation, lncRNA—long noncoding RNA, LRRK2—leucin-rich repeat kinase 2, HB—heparin-binding, LXRα—liver X receptor alpha, MASLD—metabolic dysfunction-associated steatotic liver disease, Mvb12b—multivesicular body subunit 12B, NAFLD—nonalcoholic fatty liver disease, NFAT—nuclear factor of activated T cells, NLRP3—NLR family pyrin domain-containing 3, Nron—noncoding repressor of NFAT, Nr1d1—nuclear receptor subfamily 1 group D member 1, nt—nucleotides, PER2—period circadian clock 2, PPARγ—peroxisome proliferator-activated receptor gamma, SAH—S-adenosylhomocysteine, SAHH—S-adenosylhomocysteine hydrolase, SAM—S-adenosylmethionine, SIRT1—sirtuin 1, Snhg9—small nucleolar RNA host gene 9, Snora78—small nucleolar RNA 78, Srebp1c—sterol regulatory element-binding transcription factor 1c, T2D—type 2 diabetes, TGs – triglycerides, Txnip—thioredoxin-interacting protein, Ucp1—uncoupling protein 1, VLDL—very-low-density lipoprotein, WAT—white adipose tissue, ZBTB7B—zinc finger and BTB domain-containing protein 7.
